# Augmented intelligence with voice assistance and automated machine learning in Industry 5.0

**DOI:** 10.3389/frai.2025.1538840

**Published:** 2025-03-04

**Authors:** Alexandros Bousdekis, Mina Foosherian, Mattheos Fikardos, Stefan Wellsandt, Katerina Lepenioti, Enrica Bosani, Gregoris Mentzas, Klaus-Dieter Thoben

**Affiliations:** ^1^Information Management Unit (IMU), Institute of Communication and Computer Systems (ICCS), National Technical University of Athens (NTUA), Athens, Greece; ^2^BIBA - Bremer Institut für Produktion und Logistik GmbH at the University of Bremen, Bremen, Germany; ^3^Beko Europe, Varese, Italy

**Keywords:** digital intelligent assistant, automated machine learning, voice assistance, human-AI collaboration, artificial intelligence, smart manufacturing

## Abstract

Augmented intelligence puts together human and artificial agents to create a socio-technological system, so that they co-evolve by learning and optimizing decisions through intuitive interfaces, such as conversational, voice-enabled interfaces. However, existing research works on voice assistants relies on knowledge management and simulation methods instead of data-driven algorithms. In addition, practical application and evaluation in real-life scenarios are scarce and limited in scope. In this paper, we propose the integration of voice assistance technology with Automated Machine Learning (AutoML) in order to enable the realization of the augmented intelligence paradigm in the context of Industry 5.0. In this way, the user is able to interact with the assistant through Speech-To-Text (STT) and Text-To-Speech (TTS) technologies, and consequently with the Machine Learning (ML) pipelines that are automatically created with AutoML, through voice in order to receive immediate insights while performing their task. The proposed approach was evaluated in a real manufacturing environment. We followed a structured evaluation methodology, and we analyzed the results, which demonstrates the effectiveness of our proposed approach.

## 1 Introduction

Industry 5.0 relies on placing human well-being at the center of manufacturing systems (Leng et al., 2022) and has recently been attracting the attention of researchers and practitioners in terms of both social and technological aspects (Leng et al., 2022). Human-centric manufacturing is a prerequisite for factories aiming at achieving flexibility, agility, and robustness against disruptions (Nguyen Ngoc et al., 2022; Wang et al., 2022; Bousdekis et al., 2020). From the technological perspective, enabling technologies, such as human-machine interaction, that combine the strengths of humans and machines as well as big data analytics for providing data-driven insights for advanced manufacturing systems, leading to actionable intelligence, are of outmost importance (Xu et al., 2021; Maddikunta et al., 2022).

As far as human-machine interaction is concerned, voice-enabled assistants have the potential to provide intuitive access to information and knowledge, minimize operators’ cognitive workload, and support on-the-job training (de Assis Dornelles et al., 2022; Zheng et al., 2024). Voice assistants are intent-oriented support systems that make use of an infrastructure of digital services, i.e., they target the fulfillment of user intents expressed in natural language aiming at reducing the number of interaction steps of the user (Gärtler and Schmidt, 2021). As far as data analytics is concerned, in today’s manufacturing environment, data-driven decision-making is enabled by Machine Learning (ML) algorithms, which aim to process large amounts of data in order to provide insights (Lepenioti et al., 2020). However, building an accurate ML model requires data science knowledge, which does not exist in the manufacturing workforce (Bangaru et al., 2019; Chaabi et al., 2022). Automated Machine Learning (AutoML) can overcome this challenge. AutoML aims at making ML accessible for non-ML experts (domain experts), by automating the configuration and execution of ML pipelines and models (Karmaker et al., 2021; Barbudo et al., 2023).

In the context of Industry 5.0, the integration of voice assistance and AutoML technologies can contribute to the achievement of augmented intelligence. Augmented intelligence puts together human and artificial agents to create a socio-technological system, so that they co-evolve by learning and optimizing decisions through intuitive interfaces, such as conversational, voice-enabled interfaces (Wellsandt et al., 2022). However, existing research works on voice assistants rely on knowledge management and simulation methods instead of data-driven algorithms that could take advantage of the large amounts of data existing in manufacturing enterprise systems (Bousdekis et al., 2021; Saka et al., 2023; Zheng et al., 2024; Gärtler and Schmidt, 2021). Even for these existing works, their adoption faces several barriers, thus leading to limited and unrealistic practical applications (Longo and Padovano, 2020). There is limited acceptance by operators (de Assis Dornelles et al., 2022), while such technologies require long setup periods and extensive training (Freire et al., 2022). Therefore, the practical application and evaluation in real-life scenarios are scarce and limited in scope (Longo and Padovano, 2020; Mirbabaie et al., 2021; Zheng et al., 2024), while there is a research gap on how to evaluate such solutions (Bernard and Arnold, 2019; Colabianchi et al., 2024). Only if the advantages of voice control for an efficient and secure production are sufficiently quantified, manufactures and users will consider applying such novel approaches as viable solutions (Norda et al., 2023).

The objective of this paper is to design and develop an integrated solution incorporating voice assistance technology and AutoML in order to enable the realization of the augmented intelligence paradigm in the context of Industry 5.0. AutoML automates the building and deployment of ML pipelines without requiring ML knowledge, while the voice interface exposes to the user the data analytics outcomes in an intuitive way in the context of dialogues. On the other hand, the user is able to interact with the assistant, and consequently with the ML models, through natural language in order to receive immediate insights while performing their task. The proposed approach is evaluated in a real manufacturing environment and follows a structured evaluation methodology to analyze the results.

The rest of the paper is organized as follows. Section 2 outlines the literature review on voice interfaces and AutoML approaches in manufacturing. Section 3 presents the proposed approach for augmented intelligence with voice assistance and AutoML in the frame of Industry 5.0. Section 4 implements the proposed approach in a manufacturing environment and Section 5 presents the evaluation results. Section 6 concludes the paper and presents our plans for future work.

## 2 Literature review

Voice-enabled assistants have the potential to provide intuitive access to information and knowledge, thus maximizing users’ cognitive efficiency (de Assis Dornelles et al., 2022). Despite the emergence of voice assistants in everyday life or in the service sector, and the availability of technical frameworks to create custom human-centric applications, their exploitation in the manufacturing sector is still underexplored (Ludwig et al., 2023; Mukherjee et al., 2024; Norda et al., 2023; Afanasev et al., 2019). Among others, this is due to the fact that user acceptance of voice assistants is lower than GUI-based systems, while there is the need for more effort on the development side to have a robust system (Gärtler and Schmidt, 2021). However, voice assistance technology in manufacturing has the potential to tackle with the high cognitive load in the workspace and the shortage of highly skilled workforce (Linares-Garcia et al., 2022; Ionescu and Schlund, 2021), but at the same time, it faces some distinct challenges (Ghofrani and Reichelt, 2019). For example, manufacturing operations are complex, investments in equipment are expensive, the manufacturing environment can become dangerous, the shopfloor is noisy, and the users are experts (Norda et al., 2023). These challenges, together with negative experience with voice control in the service sector prevent manufacturers from adopting voice assistance technology, also given the fact that there are not detailed quantitative evaluation approaches and results in manufacturing environments (Norda et al., 2023). During the last years, there has been an increasing research interest in assistants for manufacturing. In the literature, they are mentioned as ‘virtual assistants’, ‘Digital Intelligent Assistants’ (DIA), ‘voice assistants’, or ‘softbots’.

Jwo et al. (2021) proposed an assistant in order to facilitate the interaction between the user and the dashboard through natural language. Longo and Padovano (2020) developed a web application integrated to an ontology which adopts a flexible tree structure and a keyword labeling mechanism. Afanasev et al. (2019) presented a method and a prototype for the implementation of a voice assistant in manufacturing processes automation proposed a voice assistant as part of a Cyber-Physical System (CPS)in order to support data access automation. Li et al. (2023) presented an assistant for human-robot interaction aiming at managing several types of robots on the shop floor be embedding a pre-trained model to support the prediction of the intents. Li and Yang (2021) proposed an assistant for various manufacturing operations, such as order processing and production execution. Ruiz et al. (2023) proposed a question-answering system for supporting operators in getting access to relevant information.

Rabelo et al. (2018) presented a proof-of-concept for softbots for facilitating human-machine collaboration, sustainability of the manufacturing workforce, operational excellence, inclusiveness, satisfaction and motivation, safety, and continuous learning. Abner et al. (2020) proposed a softbot that uses data analytics and maturity models in order to support the decision-making of managers. Zambiasi et al. (2022) presented the concept of the resilient Operator 5.0, which aims at providing intuitive, human-centered, and cognitive working environments., by combining softbots and augmented reality for predictive maintenance.

Mukherjee et al. (2024) proposed a concept for voice assistants addressing the machine tools sector aiming at increasing the efficiency and safety of workers. To do this, they propose the use of speech-to-text pipelines. Norda et al. (2023) explored the use of voice interfaces in various scenarios in the CNC milling machines domain, either replacing or complementing existing touch control interactions. They found out that voice interfaces have the potential to contribute to time efficiency, especially for complex commands.

Wellsandt et al. (2020) examined a concept for a voice-enabled DIA for predictive maintenance. The authors identified the key functional modules for such an assistant as well as the requirements and constraints for its development. Wellsandt et al. (2022) proposed the adoption of a DIA for maintenance experts in order to enable the collection of feedback about the success of maintenance interventions. Colabianchi et al. (2024) proposed a DIA for manufacturing which utilizes Large Language Models (LLMs) targeted to assembly processes, and they assessed the technical robustness, the effect on operators’ cognitive workload, and the user experience of their approach in a laboratory experiment.

The aforementioned research works are either conceptual or are based upon knowledge-based methods for structuring the acquired knowledge; the use of advanced data analytics algorithms and ML models has not been investigated (Abner et al., 2020; Karmaker et al., 2021). There are several challenges on providing ML-generated insights through a voice assistant in the context of dialogues, instead of visualization-based GUIs. Moreover, the configuration of ML pipelines is a laborious and costly activity, and it becomes even more difficult when integrating a voice-enabled interface. To this end, Bousdekis et al. (2021) proposed an augmented analytics framework for implementing quality analytics integrated into a voice interface for exposing the results to the user. However, their approach is still conceptual, and it does not dive into the specificities of ML models and algorithms.

On the other hand, AutoML has been gathering increasing research attention due to its capability of making ML accessible for non-ML experts by automating all the ML stages (Barbudo et al., 2023), and of usually outperforming conventional ML algorithms (Liang and Xue, 2023). The AutoML paradigm aims to automate the ML aspect of real-world applications through an end-to-end process (Karmaker et al., 2021). Karmaker et al. (2021) proposed six levels of automation for the different AutoML systems, each with varying automated tasks and accessibility to domain experts. Furthermore in the literature, there is a plethora of pipelines and methodologies that compromise AutoML. He et al. (2021) provide a pipeline that incorporates four main steps: (i) Data Preparation, (ii) Feature Engineering, (iii) Model Generation and (iv) Model Evaluation, where each one has multiple sub-tasks. The Model Generation step can be split into two sub-steps: the Search Space and the Optimization Methods. The former defines the design of the ML models (traditional or neural networks), while the latter handles hyperparameter optimization and architecture optimization (AO). These two sub-steps are the first to be automated from the aforementioned automation levels and are of great interest due to their increased computational and resource intensity. Researchers experimented with different methodologies to find optimal ML models and counterbalance the computational needs. For the hyperparameter optimization these methods adopt approaches such as Grid Search, Random Search, Bayesian Optimization, Early-stopping and Multi-fidelity Optimization (Yu and Zhu, 2020, Hutter et al., 2019). In addition, Nikitin et al. (2022) proposed FEDOT, which designs composite ML pipelines, through model and data operations that create a directed acyclic graph and automate them through an evolutionary approach and hyperparameter optimization. Despite the merits of automating the ML aspects, AutoML also has limitations regarding real-world applications, specifically with the available budget of time and computation. The hyperparameter optimization can be computationally expensive (He et al., 2021), and the whole AutoML framework adopted can result in a long waiting time to find solutions (Elshawi et al., 2019). The trade-off between time and computational resources creates strategic decision-making needs to balance the respective constraints (Azevedo et al., 2024). Nevertheless, the advantages of AutoML have made available several approaches and applications in the manufacturing realm. It is noteworthy that most of the existing research on AutoML in manufacturing deals with maintenance and quality operations. This is due to the fact that both operations include many non-value-adding activities that contribute significantly to the manufacturing firms’ costs. However, although maintenance has largely adopted sensory technology in order to automate decision-making, quality procedures remain, to a large extent, manual in nature.

Schuh et al. (2022) provided a list of requirements and use cases that manufacturing companies should take into account when selecting an AutoML solution. Gerling et al. (2022) proposed an AutoML-based system that generates predictions about production faults and indications about the related root causes. Chaabi et al. (2022) applied AutoML methods in various manufacturing applications, such as quality control and predictive maintenance. Mallouk et al. (2023) presented an AutoML-based approach that aims at providing decision support to manufacturing experts. Krauß et al. (2020) performed a comparative analysis of AutoML frameworks in manufacturing, by also comparing it to manual processes for quality control. Zhai et al. (2023) proposed a domain-specific AutoML approach for anomaly detection and defect diagnosis in the semiconductor industry. Jayasurya et al. (2024) proposed a framework based on AutoML, employing tools such as AutoKeras, Sweetviz, NumPy, pandas, Streamlit, and PyCaret, in the context of predictive maintenance. Conrad et al. (2024) developed a workflow in the production engineering domain with the use of AutoML and compared it with the manual data mining process. Rooney et al. (2024) used AutoML for failure detection in additive manufacturing. Denkena et al. (2020) demonstrated the improvements achieved by AutoML to predict shape errors during milling for cold rolling procedures. Sousa et al. (2022) used the CRISP-DM methodology and AutoML to address production time prediction for metal containers production. They compared four open-source modern AutoML technologies: AutoGluon, H2O AutoML, rminer, and TPOT. Fikardos et al. (2022) proposed a framework architecture that utilizes AutoML in predictive quality.

AutoML has been proved to achieve a competitive performance; however, apart from technical challenges, related to, e.g., features selection and class imbalance (Zhai et al., 2024; Salehin et al., 2024), recent studies have shown that the lack of transparency and explainability of AutoML make the users reluctant to trust them (Crisan and Fiore-Gartland, 2021; Drozdal et al., 2020; Wang et al., 2021). Existing transparency and explainability approaches incorporate interactive visualization techniques (Zöller et al., 2023; Garouani et al., 2022; Amirian et al., 2021) and post-hoc explanation methods (Zhai et al., 2024). Although such approaches have been proved promising for GUI-based systems, they are not suitable when integrating AutoML to voice interfaces.

In the context of Industry 5.0, the integration of voice assistance and AutoML technologies can contribute to the achievement of augmented intelligence. However, such an approach has not been investigated, although it has the potential to enable and augment operators with data-driven insights in an intuitive, human-like, and ‘hands-free’ way, without requiring ML expertise, thus increasing the efficiency of their work.

## 3 The proposed solution for augmented intelligence with voice assistance and automated machine learning

**Figure 1:**
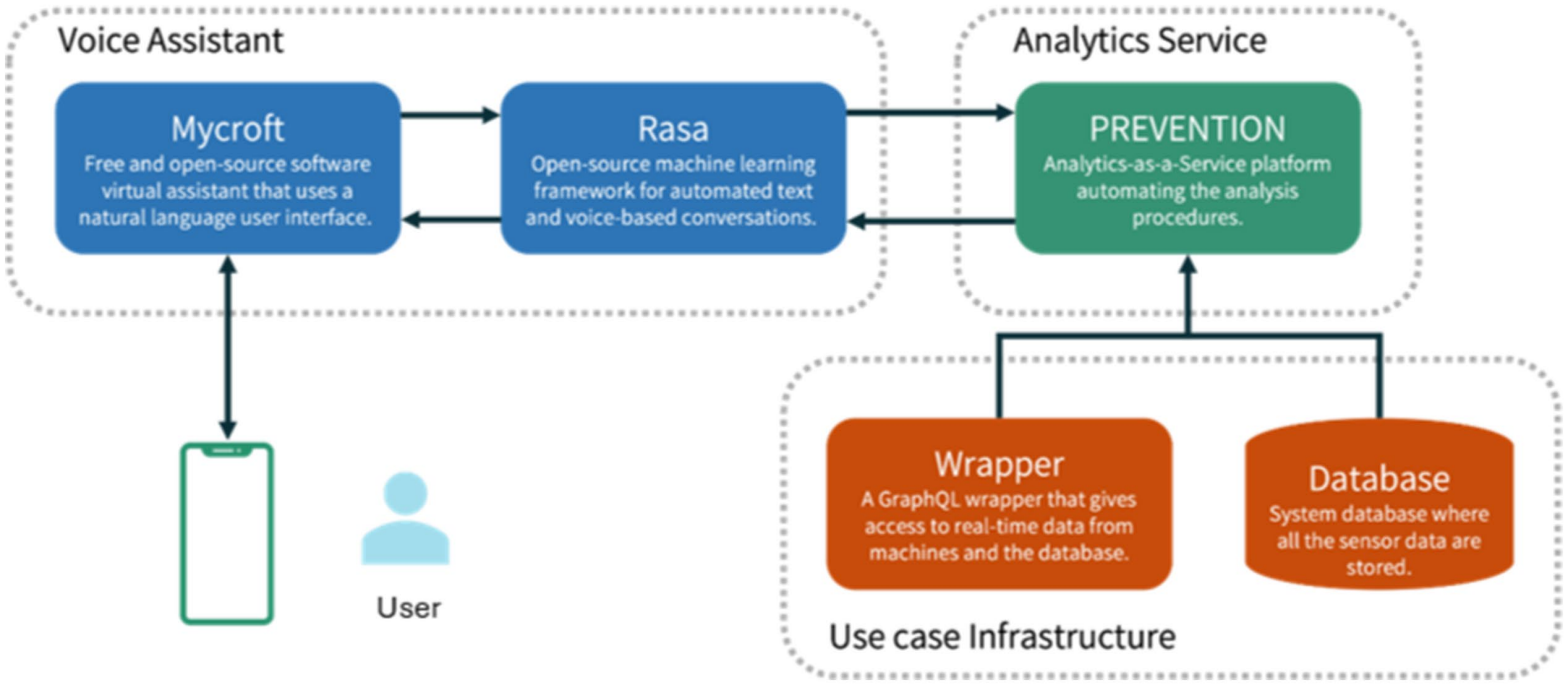
High-level architecture.

The high-level architecture for augmented intelligence with voice assistance technology and AutoML is depicted in Figure 1. It embeds three main modules: Voice Assistant, Analytics Service, and Use Case Infrastructure. The Voice Assistant module corresponds to the DIA core and manages user interactions. This module first captures the user’s message via the Android app and passes the message to the Conversational Agent Team (CAT), which is the technical integration of a Mycroft skill and a Rasa chatbot to minimize the disadvantages of the individual agents. We use Mycroft as the leading agent because users begin all their dialogues through it. The second agent is a Rasa chatbot—both exchange text messages, but only Mycroft responds to users directly. Both Mycroft and Rasa are open-source chatbot frameworks. However, Rasa facilitates sophisticated support in developing Natural Language Understanding (NLU) and Dialog Management (DM). Rasa uses a pipeline for NLU that can integrate various open NLU components, such as Spacy or Duckling. It applies a hybrid approach for DM, combining rules (reliable) with probabilistic models (flexible). Developers can train the latter based on real conversations and thus continuously improve the assistant. In our solution, Mycroft passes user requests to Rasa, where the request is mapped to one of the defined intents of the assistant, and a query is formulated. Rasa’s responsibilities are to interpret the user input and generate the requests as queries for the Analytics Service, which responds with the insights needed. The Analytics Service communicates directly with the use case infrastructure, retrieving all the necessary data and information required to make the analyses and produce the outcomes. It also processes the received query, and the results of the requested analytics are encapsulated into a response sent back to the DIA. These modules are further detailed in the following sub-sections.

### 3.1 Voice assistant

**Figure 2:**
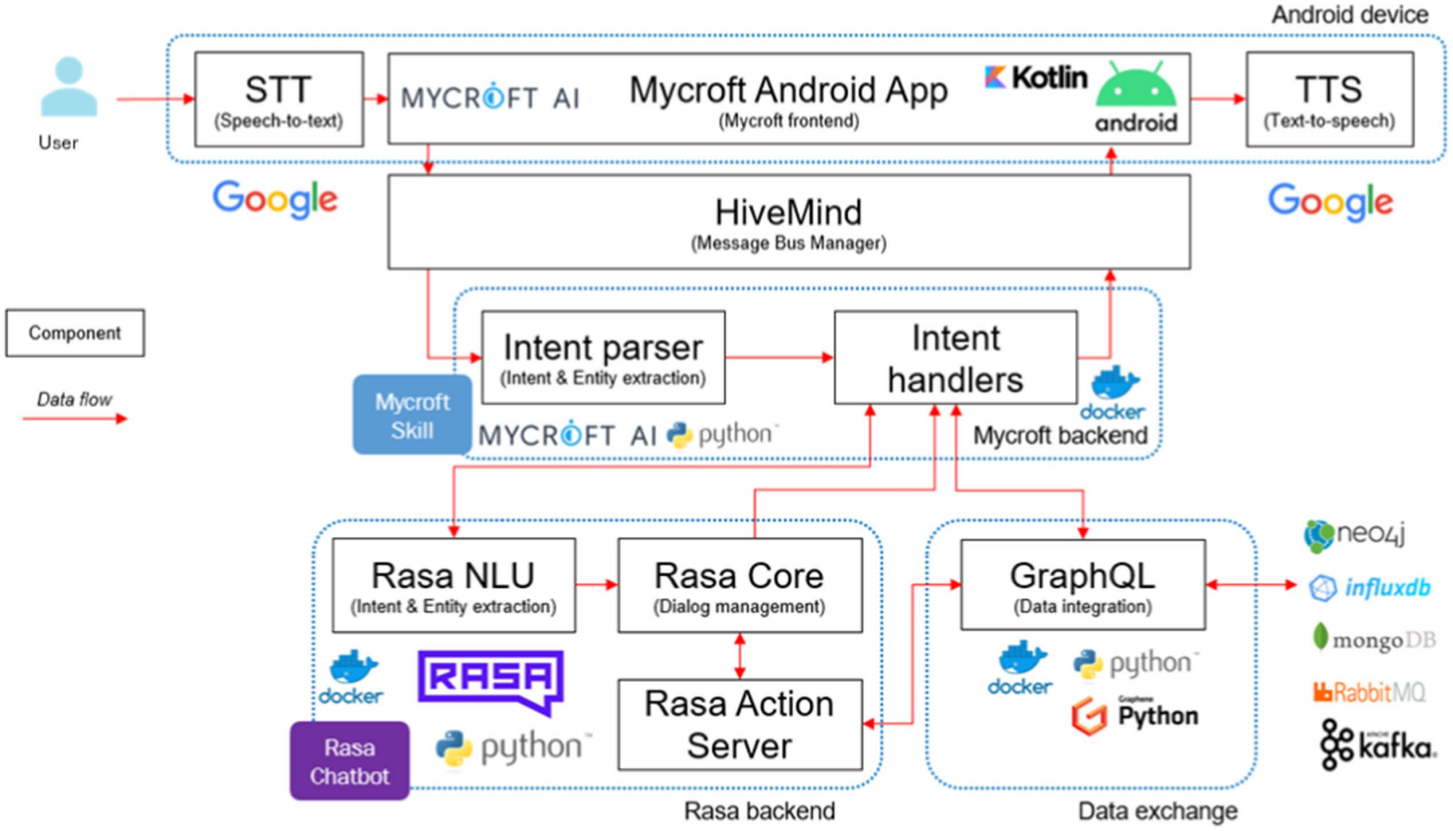
DIA core architecture.

The interface with which the user interacts, as well as the link between the analytics component and the user, is the DIA core, which covers the Android App, the message bus manager, Mycroft, the “Talk to Rasa” skill (in Mycroft), the Rasa chatbot, and the data exchange. The information flow among the various components of the DIA core is depicted in Figure 2.

Users interact with Mycroft through the Android app, where Google Speech-To-Text (STT) transcribes audio to text. The Mycroft core is accessible via a reverse proxy supporting secure connections managed by security mechanisms. Integration with Keycloak ensures that only authorized users can access Mycroft, with the app exchanging valid tokens with Keycloak for authentication. HiveMind, running as a separate Docker container, adds a security and management layer, enabling multi-user interactions and extending Mycroft core to various devices, including those not running Mycroft natively.

Mycroft’s Natural Language Understanding (NLU) identifies user intents and entities from transcribed utterances and tries to match the intent with a suitable skill. Here we have a customized “Talk to Rasa” skill that facilitates message exchange between Mycroft and Rasa. This way, complex dialogs are managed by Rasa, with a configurable NLU pipeline, a dialog manager based on rules and probabilistic models, and a fulfillment server performing custom actions via Python code. Rasa fulfills user intents by executing code and accessing external data sources. Rasa action server uses the data exchange services responses (i.e., semi-structured data from analytics service) to build a human-readable response message, which is sent to the user via Mycroft. The Google Text-to-Speech (TTS) service generates audio from text in the Android app.

### 3.2 Analytics service

**Figure 3:**
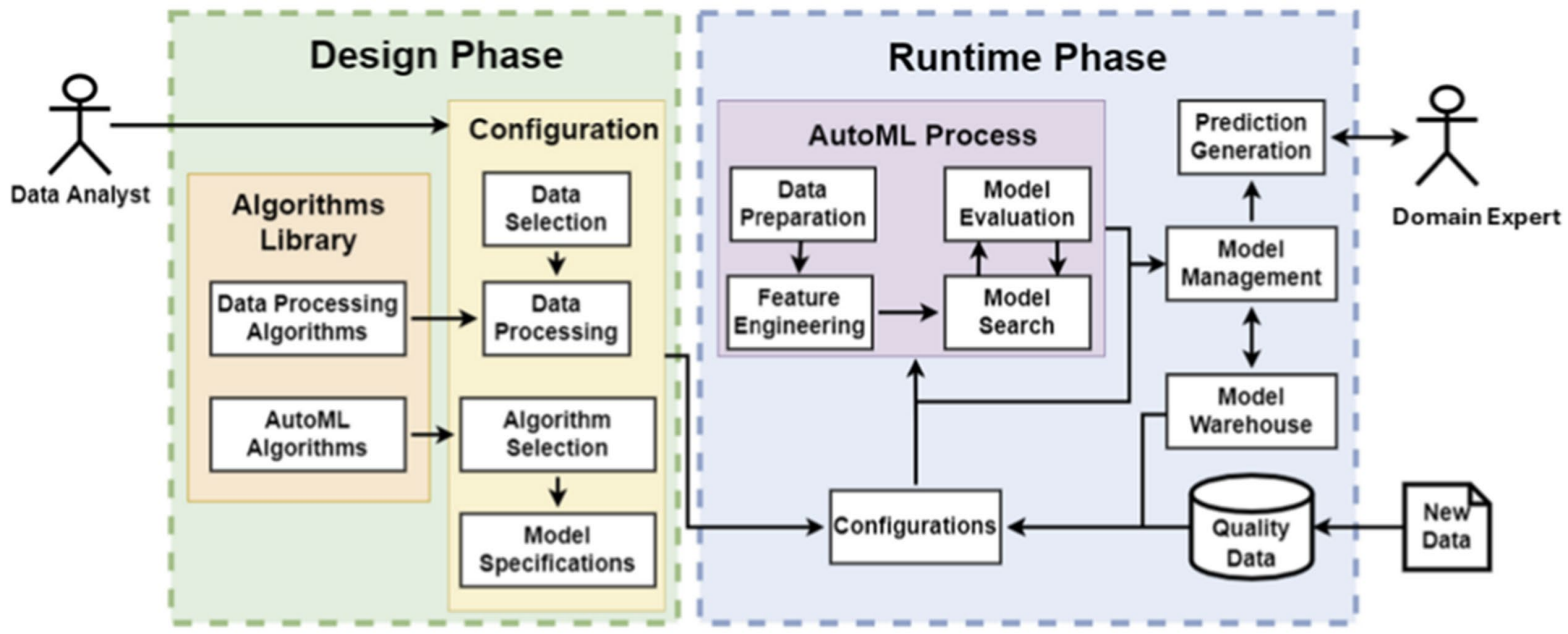
Analytics service architecture for AutoML.

The Analytics Service takes advantage of the AutoML process in order to minimize human intervention while constructing and configuring ML within specific computational limits. In this way, it tackles effectively with the challenge of developing appropriate ML models for the problem at hand since the ML model suitability depends on the available dataset, its preprocessing, as well as the configuration of algorithms’ parameters. Further, these computing skills and ML knowledge do not usually exist in the manufacturing workforce. The architecture of the Analytics Service is distinguished to the Design Phase and the Runtime Phase, as depicted in Figure 3.

In the Design phase, the data analyst selects and configures the appropriate AutoML frameworks. In the Configuration component of the Design phase, the data analyst applies Data Processing Algorithms, retrieved from the Algorithms Library, on the dataset in order to pursue data cleaning and feature engineering. Then, he/ she defines and configures the AutoML framework to be used with regards to model parameters, evaluation metrics, and termination conditions. Any further configurations, such as model acceptance conditions and output formats, can be defined in the Model Specifications.

In the Runtime phase, the AutoML process is executed as soon as the data analyst defines new configurations or as soon as new data becomes available for the already configured AutoML models. In the first case, the algorithm evaluates several models and optimizes the candidate ones. The finally selected model feeds into the Model Management component in order either to be stored in the Model Warehouse or to be discarded according to the acceptance conditions that have been configured in the Design phase. In the second case, the models are automatically retrained or optimized by incorporating the new data that has become available. They are retrieved from the Model Warehouse in order to feed into the AutoML process. The new model feeds into the Model Management process in order to compare its performance with the one of the previous model. The best-performing model is stored, and the other one is discarded.

### 3.3 Use case infrastructure

The Voice Assistant and Analytics Service technologies need to be integrated to the use case infrastructure in order to store and retrieve data that are required for the analyses. This data can be accessed through APIs or directly from the database.

## 4 Deployment in quality control operations

In this Section, we describe the use case under examination, which is the quality control in the home appliances industry (Section 4.1), and we present some indicative demonstration scenarios of the proposed solution (Section 4.2).

### 4.1 Home appliances use case

**Figure 4:**
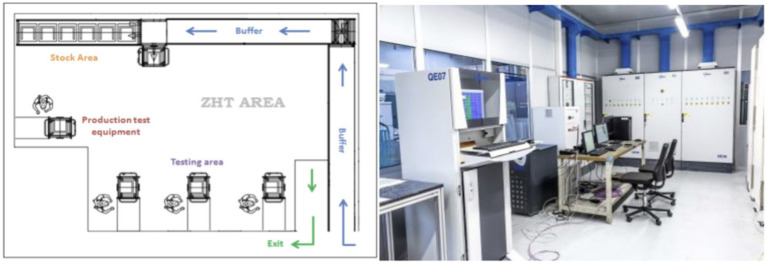
Zero hour testing (ZHT) laboratory at whirlpool.

The use case under examination concerns the quality control procedures of Whirlpool, one of the leading companies in the home appliances industry, with around 92,000 employees and over 70 manufacturing and technology research centers worldwide. The use case addresses the end-of-line quality, which involves quality testing in order to ensure a high standard level of product quality to final customers. To these testing actions, the Whirlpool Production system also adds some statistical quality check actions that are applied both on internal production parts on quality critical processes (statistical process control stations) and on finished goods after the packaging process. In particular, this last testing, called Zero Hour Testing (ZHT) (Figure 4) refers to the Statistical Quality Control applied in a dedicated laboratory out of production flow on some finished products retrieved from the quantities ready to be delivered to the markets. The main objectives of ZHT are to measure the quality level of the outgoing product from an aesthetic, functional, and normative point and to measure the effectiveness of process control. These tests are executed in a dedicated laboratory environment, created in each production site, and following a specific operating procedure. This testing method is designed to replicate the customer approach to the product, simulating the normal product usage conditions at the final customer’s first usage.

### 4.2 Demonstration scenarios

**Figure 5:**
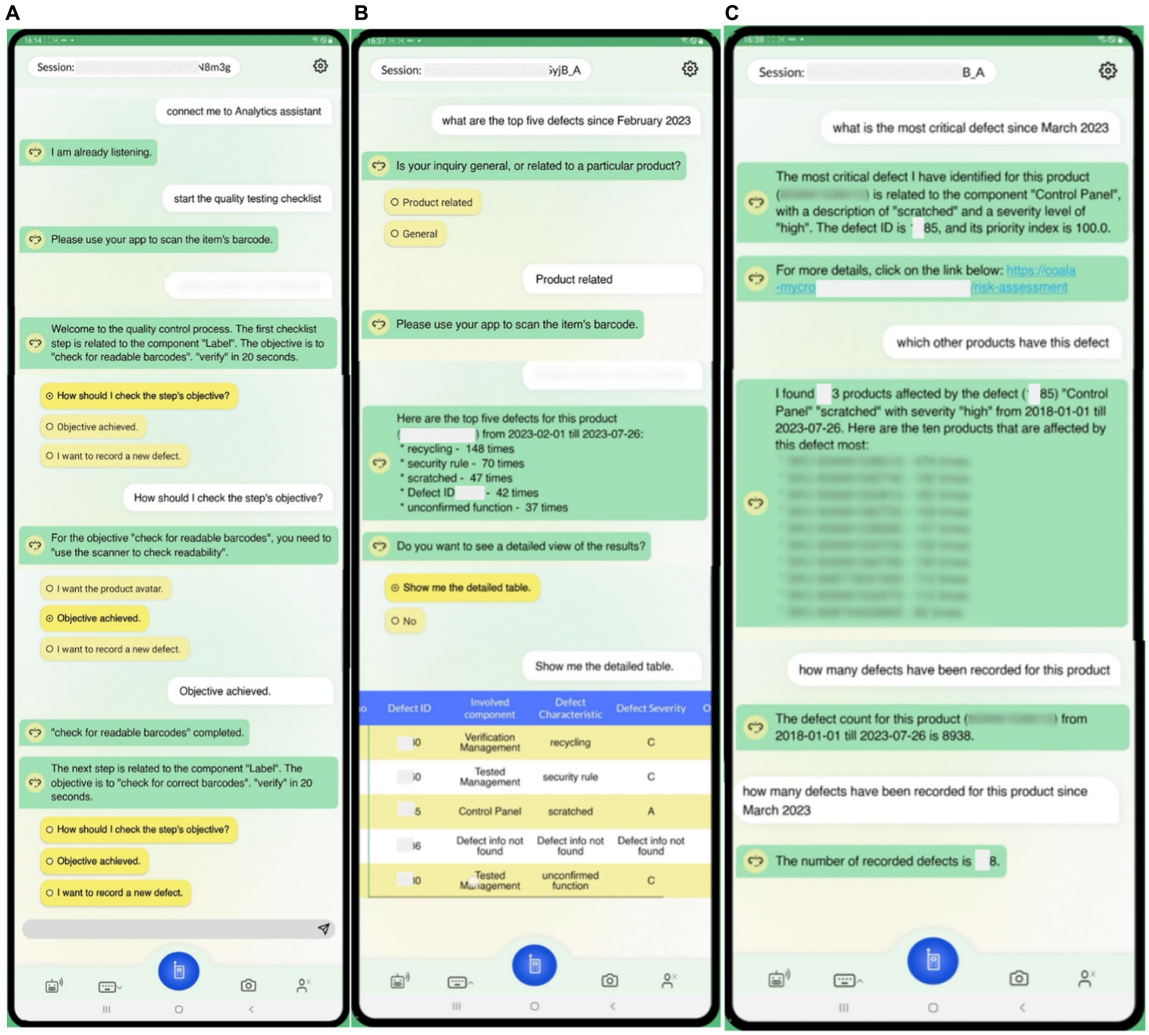
Demonstration of dialogues for end-of-line testing procedures (1). **(A)** End-of-line testing process execution in the left image, **(B)** Requesting top 5 defects with SKU and date filter in the middle image, **(C)** Requesting most critical defect with SKU and date filter, requesting products affected by a specific defect, requesting number of defects with SKU filter in the right image.

**Figure 6:**
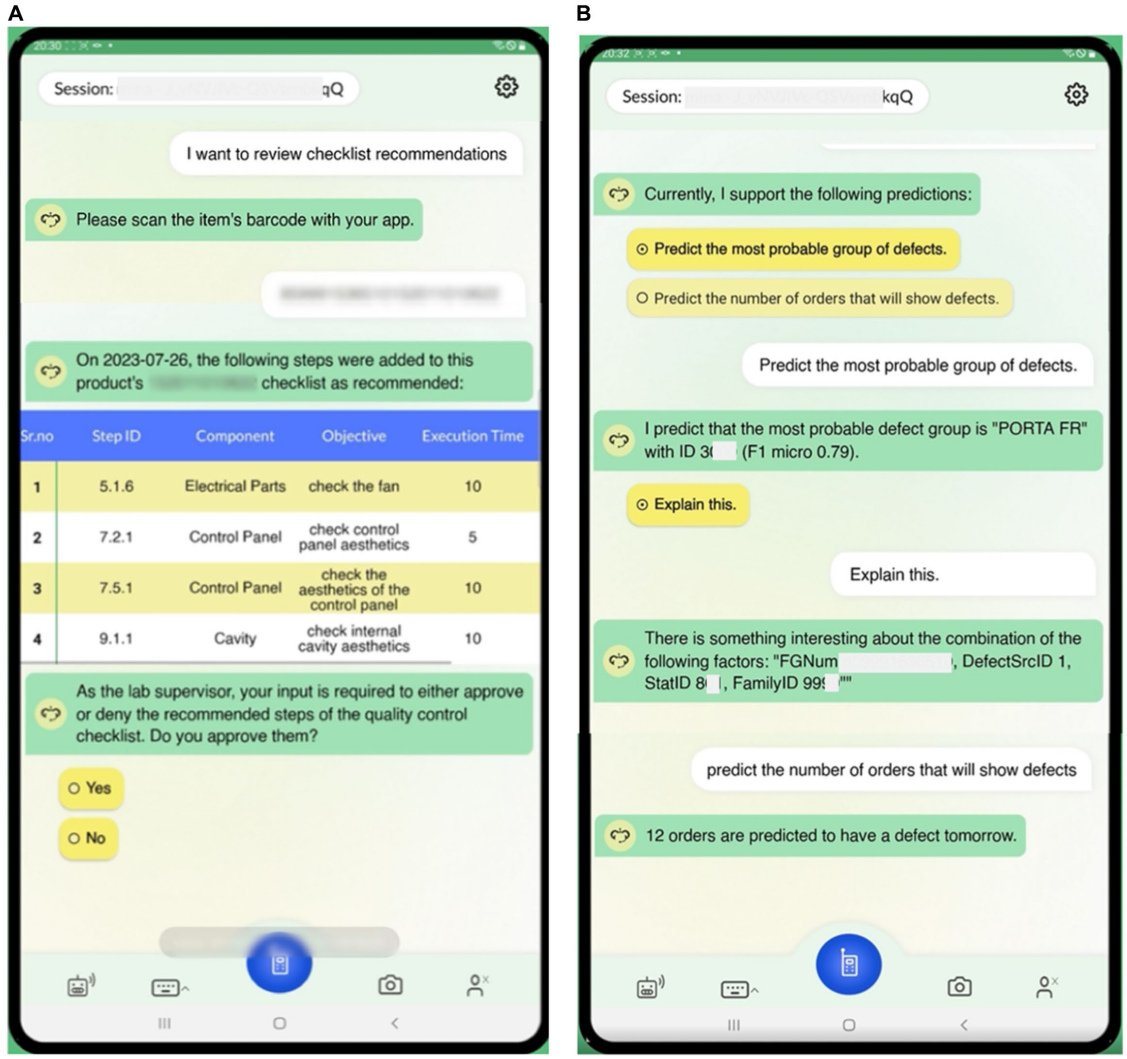
Demonstration of dialogues for end-of-line testing procedures (2). **(A)** End-of-line testing process update recommendations based on critical defect occurrences in the left image, **(B)** Requesting predictions of the most probable defect group and number of orders with defect in the next day in the right image.

In this sub-section, we demonstrate indicative scenarios in the context of the aforementioned use case. The interaction can be done via voice or text input, and the dialogues are displayed on a tablet screen in order to, among others, investigate additional product-related information and provide visualization capabilities, e.g., in case the user needs additional explanations on the generated outcomes of the algorithms. Figures 5, 6 demonstrate indicative dialogues between the user and the DIA during the end-of-line testing procedures, including the quality testing process and various analytics questions. The end-of-line quality testing process is designed to support operators with three expertise levels: novice, intermediate, and expert. Novice users receive more detailed instructions to perform the test, whereas experts receive high-level instructions. At each step, users can report a defect, and the DIA makes suggestions to assist with the defect recording process (Figure 5A).

In Tables 1, 2, we also present two example queries that facilitate the interaction of AutoML with the voice interface. In Table 1, to predict the number of orders that will manifest some defect in the following day, the user triggers the implemented analysis with the goal “PREDICT_NUMBER_OF_DEFECTS.” This analysis has been implemented using AUTOKERAS (created and trained) AutoML library. The prediction of the number of orders with defects in the following day can be used by the workers to get a glimpse of the work ahead. It can be used as an alarm mechanism for a failure in the production line, causing a greater number of defects than expected. An example conversation with the DIA using this query is presented in Figure 6B. In Table 2 the user wants to get information about the products affected by the defect with defect ID 3035, and the digital intelligent assistant is expected to return the list of the SKUs of the affected products. This scenario has been implemented by the analysis with analytics goal “GROUP_DEFECTS_BY_DEFECT_ID_AND_SKU,” using the historical data of the recorded defect occurrences. In this case, the resultRequest query with the appropriate request and filters will return the list of affected products, where each record is described by three fields: the DefectID, the FGNum (or SKU), and the count. All the records have the requested Defect ID, a unique FGNum, and the number of recorded defects for the product (count). Based on the answer, the user can understand that multiple products are affected by the given Defect ID with varying recorded occurrences. An example conversation with the DIA using this query is presented in Figure 5C.

**Table 1:** Example next day number of orders with defect prediction.

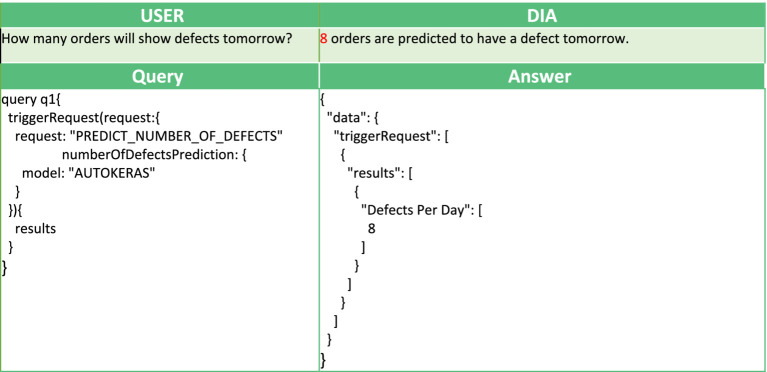

**Table 2:** Example requesting the products affected by a specific defect.

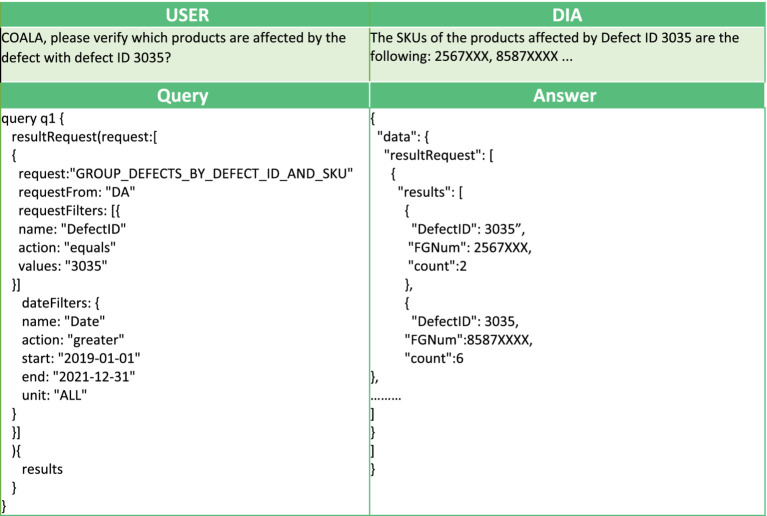

## 5 Evaluation in real-life manufacturing scenarios

In this Section, we describe the evaluation procedure and results in real-life manufacturing scenarios, in the context of the aforementioned home appliances industry. More specifically, we present our evaluation methodology for voice-enabled AI solutions (Section 5.1), the evaluation setup (Section 5.2), the evaluation results (Section 5.3), and a discussion on the generalizability criteria that need to be considered (Section 5.4).

### 5.1 Evaluation methodology

Our evaluation methodology was based on the evaluation methodology for voice-enabled AI solutions, proposed by Bousdekis et al. (2022). In our adaptation, the methodology is structured across five dimensions: AI trustworthiness, system usability, cognitive workload, technical robustness, and lessons learned. Table 3 shows the methods and tools that were used to address each dimension. Below, we briefly present the methods that address these dimensions.

**Table 3:** Method/Tool per each evaluation dimension.

Dimension	Method/Tool
AI Trustworthiness	ALTAI
System usability	SUS, VUS
Cognitive workload	NASA-TLX
Technical robustness	ML performance metrics, intent recognition accuracy
Lessons learned	Workshops, interviews

#### 5.1.1 AI trustworthiness

The concept of Trustworthy AI (TAI) dictates that humans, organizations, and societies will achieve the full potential of AI if trust can be established in its development, deployment, and use (Mentzas et al., 2024; Bousdekis et al., 2022). In this realm, the High-Level Expert Group on Artificial Intelligence (AI-HLEG) has created the Assessment List for Trustworthy Artificial Intelligence (ALTAI) tool that helps organizations to self-assess the trustworthiness of their AI systems through a questionnaire (Artificial Intelligence, 2019). The ALTAI is structured according to seven high-level requirements for TAI: Human agency and oversight; Technical robustness and safety; Privacy and data governance; Transparency; Diversity, non-discrimination and fairness; Societal and environmental well-being; Accountability.

#### 5.1.2 System usability

The usability of voice-enabled AI solutions is evaluated through System Usability Scale (SUS) (Brooke, 1996) and Voice Usability Scale (VUS) (Murad et al., 2019). Both tools are needed due to the distinct characteristics of voice interfaces, which include: understanding of pauses during a conversation (Zwakman et al., 2021), limitations to back-and-forth navigation (Holmes et al., 2019), not having a visualization of results (Cowan et al., 2017), user expectations about the structure of dialogues (Murad et al., 2019), absence of familiarity with synthetic voice (Babel et al., 2014). Both tools include 10 items having declarative statements of opinion to which the participants will respond with their rate of agreement on a Likert scale.

#### 5.1.3 Cognitive workload

The extent of human cognitive resources utilization is called cognitive load (Ninomiya et al., 2024). The emergence of AI technologies in the manufacturing domain dictates the capability of adaptation to the new processes in terms of, among others, the efficient management of workload (Matt et al., 2015). The cognitive workload can be measured through subjective measures in order to overcome the challenges in assessing operators’ performance in complex and automated environments (Rubio et al., 2004). This dimension is addressed by the NASA-TLX (Hart, 1988), a widely used subjective method, which includes six dimensions: Mental Demand; Physical Demand; Temporal Demand; Overall Performance; Effort; Frustration Level.

#### 5.1.4 Technical robustness

Technical robustness is evaluated for the two main modules of the proposed solution, i.e., Analytics Service and Voice Assistant. Regarding the Analytics Service, its AutoML models are validated by using appropriate performance metrics. It should be noted that the selection of the evaluation metrics according to the algorithm used can be automated by the AutoML process. Regarding the Voice Assistant, its performance is measured based on intent recognition accuracy for the recorded conversations.

#### 5.1.5 Lessons learned

Based on workshops among the involved users and bilateral interviews, including also the management, in this dimension, we collect qualitative feedback at the end of the evaluation procedure in order to gather the business perspective and to conclude with lessons learned for the technological solution and its adoption by the manufacturing firm.

### 5.2 Evaluation setup

**Figure 7:**
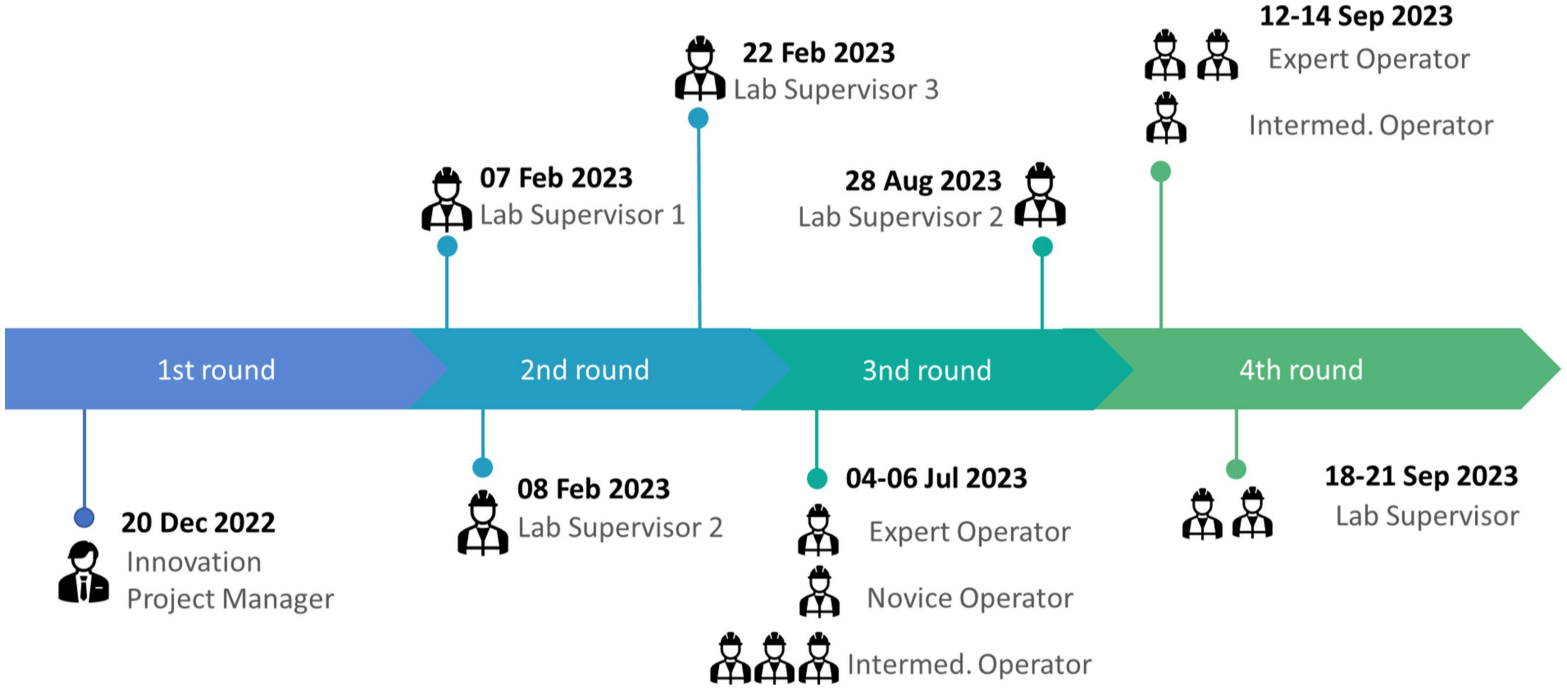
The timeline of the evaluation procedure in Whirlpool.

The evaluation procedure started on December 2022 and was being performed in parallel with the integration activities in order to continuously provide early feedback on the technical improvements. We separated the evaluation procedure in 4 rounds, engaging various roles of testers in the Whirlpool use case, as it is shown in Figure 7. During the 3rd round, we captured the last points to improve the solution, such as errors in response translations, data accuracy and quality in the quality control checklist database, etc., and we also provided training to the operators. It should be noted that the number of participants and their roles were subject to restrictions derived from the factory operations. The final evaluation was performed with 2 lab supervisors, 3 expert operators and 1 intermediate operator.

**Figure 8:**
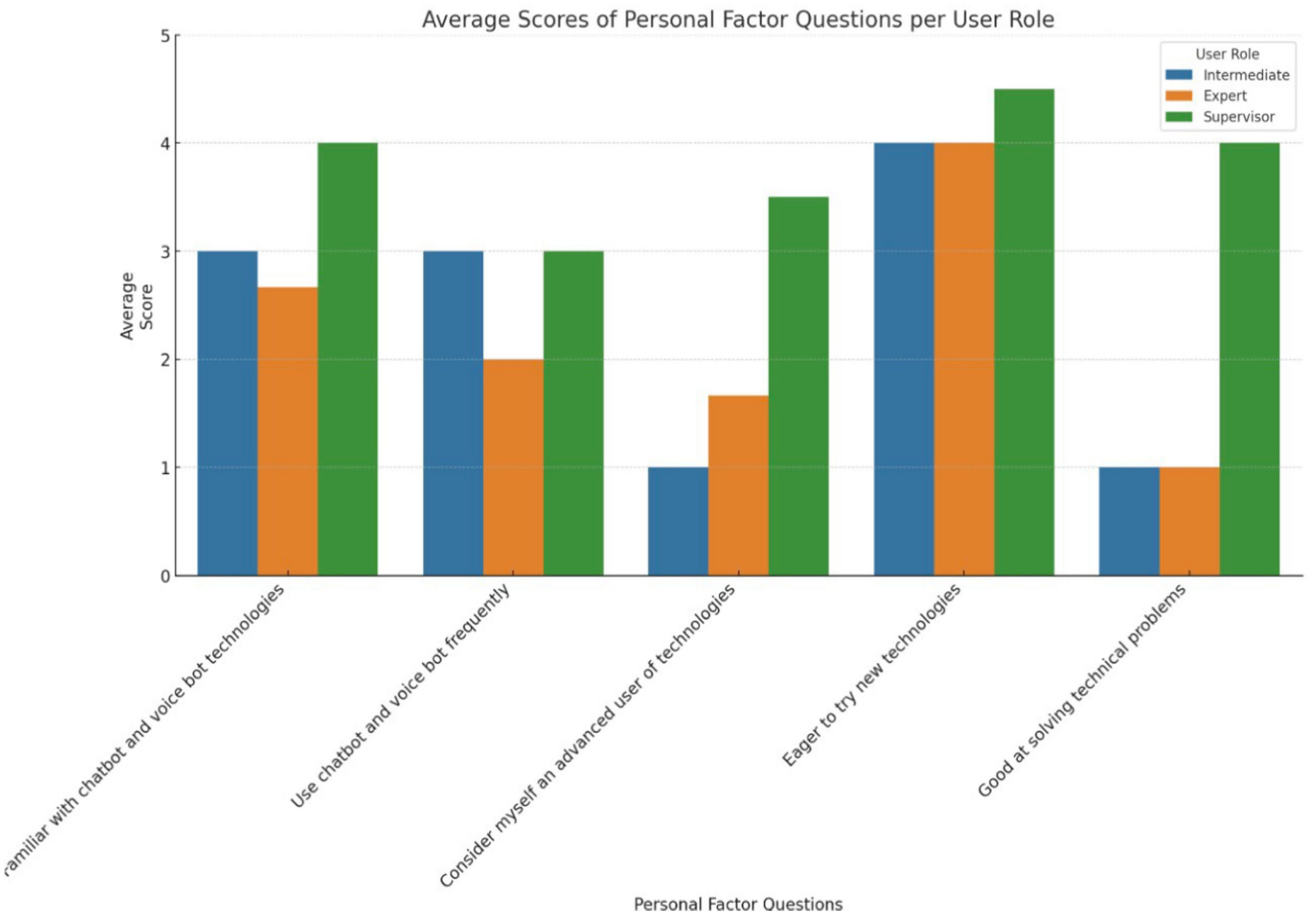
Personal factors questions per user role in Whirlpool.

During the third and fourth evaluation rounds, the procedure consisted of several key steps. Initially, participants were required to sign a printed consent form to acknowledge their participation. Subsequently, in the case of the third evaluation round, participants underwent training, which involved the use of training materials and a personalized training session using the app. The participants were asked to go through an online questionnaire that involved the following sections: user role, a set of tasks to perform based on the user’s role, NASA-TLX questionnaire, SUS questionnaire, VUS questionnaire, personal factors questionnaire including items such as orientation towards using new technologies, gender, age, education, occupation, and work experience, two additional open questions regarding trust in the technological solution. Figure 8 depicts the average scores on the Likert scale (1–5) of personal factor questions per user role.

### 5.3 Evaluation results

In this sub-section, we present the evaluation results per dimension of the evaluation methodology, i.e., AI trustworthiness (Section 5.3.1), system usability (Section 5.3.2), cognitive workload (Section 5.3.3), technical robustness (Section 5.3.4), and lessons learned (Section 5.3.5).

#### 5.3.1 AI trustworthiness

**Figure 9:**
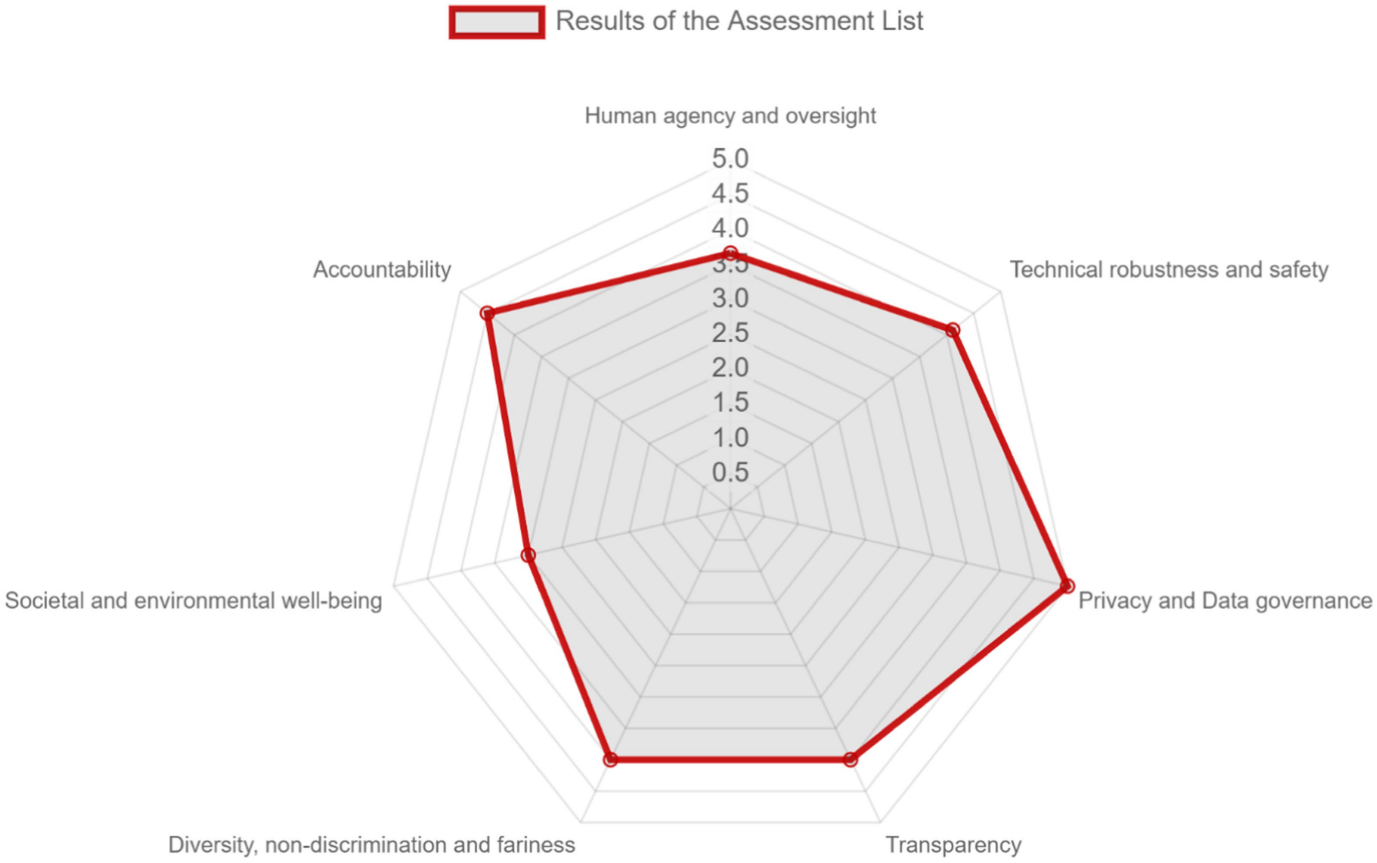
The results of the 7 requirements score as derived from ALTAI.

AI trustworthiness was assessed by a multidisciplinary team of people with both business and technical background by using the web-based tool developed by EC.^1^ After completing the web-based questionnaire, based on the responses, the tool extracts a visualization of the self-assessed level of adherence of the AI system with the TAI requirements, and recommendations. Figure 9 depicts the results in the form of a Polar diagram for the seven requirements for Trustworthy AI, while Supplementary Table S1 presents the resulting recommendations per requirement.

Overall, the solution excels in “Privacy and Data Governance,” while it performs very well with regards to “Accountability,” “Technical Robustness and Safety,” “Transparency,” and “Diversity, Non-discrimination and Fairness.” The dimensions “Human Agency and Oversight” and “Societal and Environmental Well-being” gather a lower score, although no recommendations are generated by the tool. This may be caused by some limitations of ALTAI, having been identified in the literature (Radclyffe et al., 2023; Stahl and Leach, 2023), such as the fact that some questions are not applicable in all the application domains or the candidate responses that are provided do not accurately represent its status, but they affect the resulting score.

Regarding the recommendations that ALTAI provides, one should take into account its limitations that have been already mentioned in the literature (Bousdekis et al., 2024). ALTAI has been designed for end products; it does not address the various phases of software development lifecycle. It incorporates generic questions aiming at addressing every AI system; however, the AI system may refer to a business environment with expert and qualified users. In addition, ALTAI considers as “AI system” the software and does not treat it as a socio-technical system, potentially leading to disregard of unforeseen challenges. For some questions, no alternative response is accurate, while its current structure hinders its applicability.

#### 5.3.2 System usability

The assessment of system usability was undertaken by determining the SUS and VUS scores, which were derived from the questionnaires completed by the users. Each scale encompassed a section of 10 questions, which are cited in Table 4. The findings for each scale are depicted through three bar plots representing the average scores per user, per question, and per user role. The plots that illustrate the scores per question are scaled from 1 to 5, whereas the remaining plots feature percentile scores ranging from 0 to 100.

**Table 4:** SUS items.

Code	SUS Items
SUS_1	I think that I would like to use this system frequently.
SUS_2	I found the system unnecessarily complex.
SUS_3	I thought the system was easy to use.
SUS_4	I think that I would need the support of a technical person to be able to use this system.
SUS_5	I found the various functions in this system were well integrated.
SUS_6	I thought there was too much inconsistency in this system.
SUS_7	I would imagine that most people would learn to use this system very quickly.
SUS_8	I found the system very cumbersome to use.
SUS_9	I felt very confident using the system.
SUS_10	I needed to learn a lot of things before I could get going with this system.

**Figure 10:**
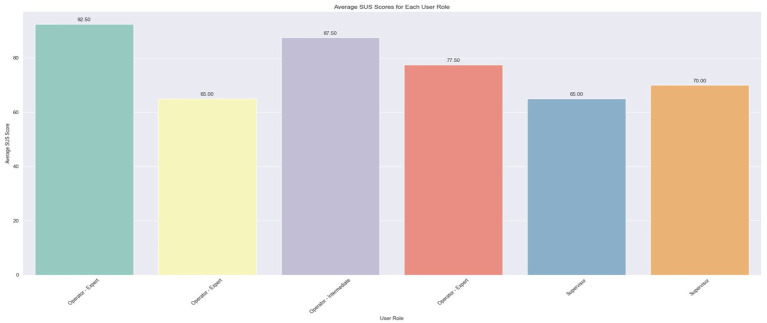
Average SUS score per user.

Figure 10 presents the average scores per user. The findings reveal that the system earned an ‘Excellent’ rating (>80.3) from two users and a ‘Good’ rating (68–80.3) from another two, while the remainder assigned it a ‘Poor’ rating (51–68). A notable variation is evident among the Expert Operators, with scores ranging from 65 to 92.5, whereas the scores of the two Supervisors are more closely aligned.

**Figure 11:**
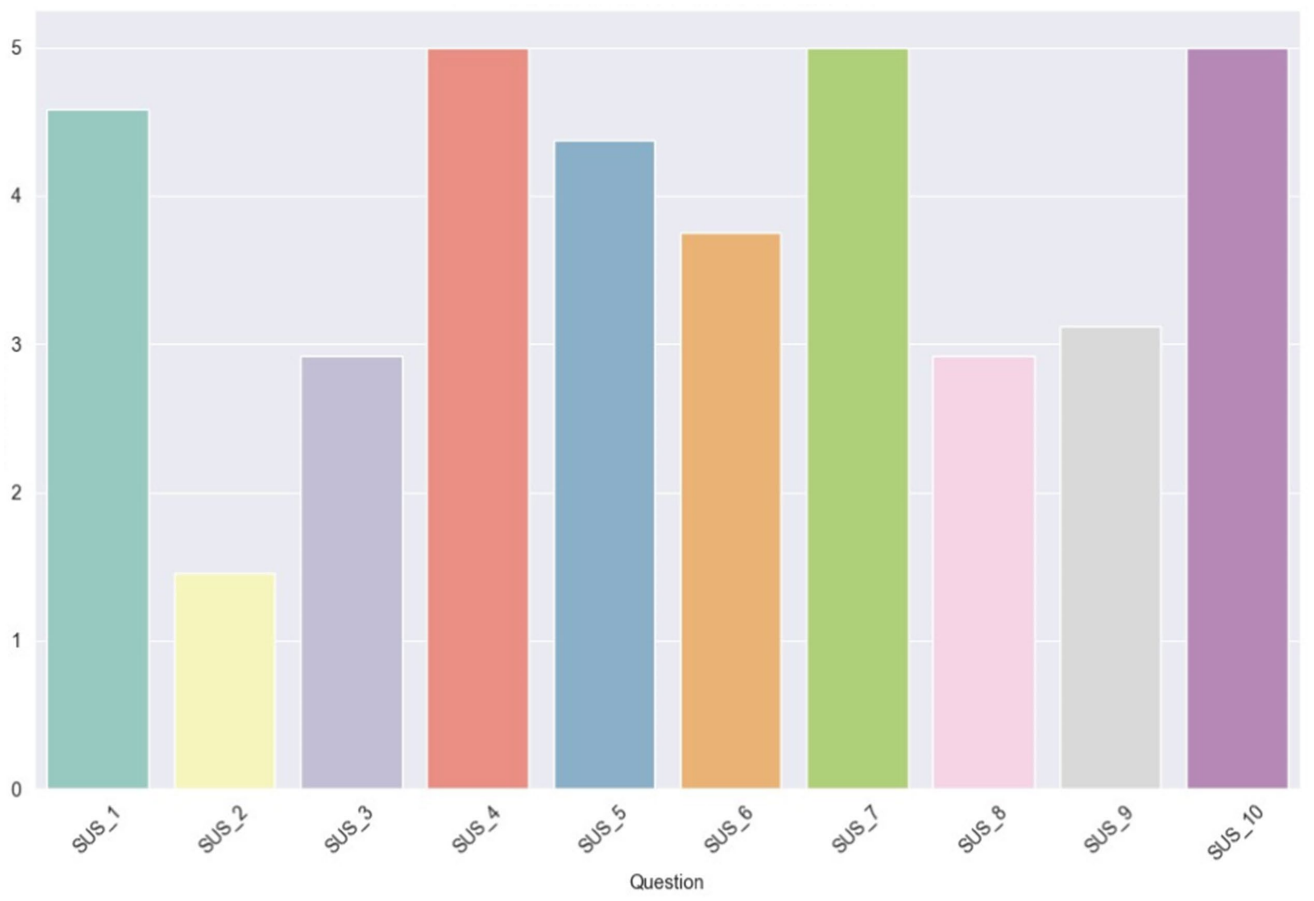
Average scores for SUS questions.

**Figure 12:**
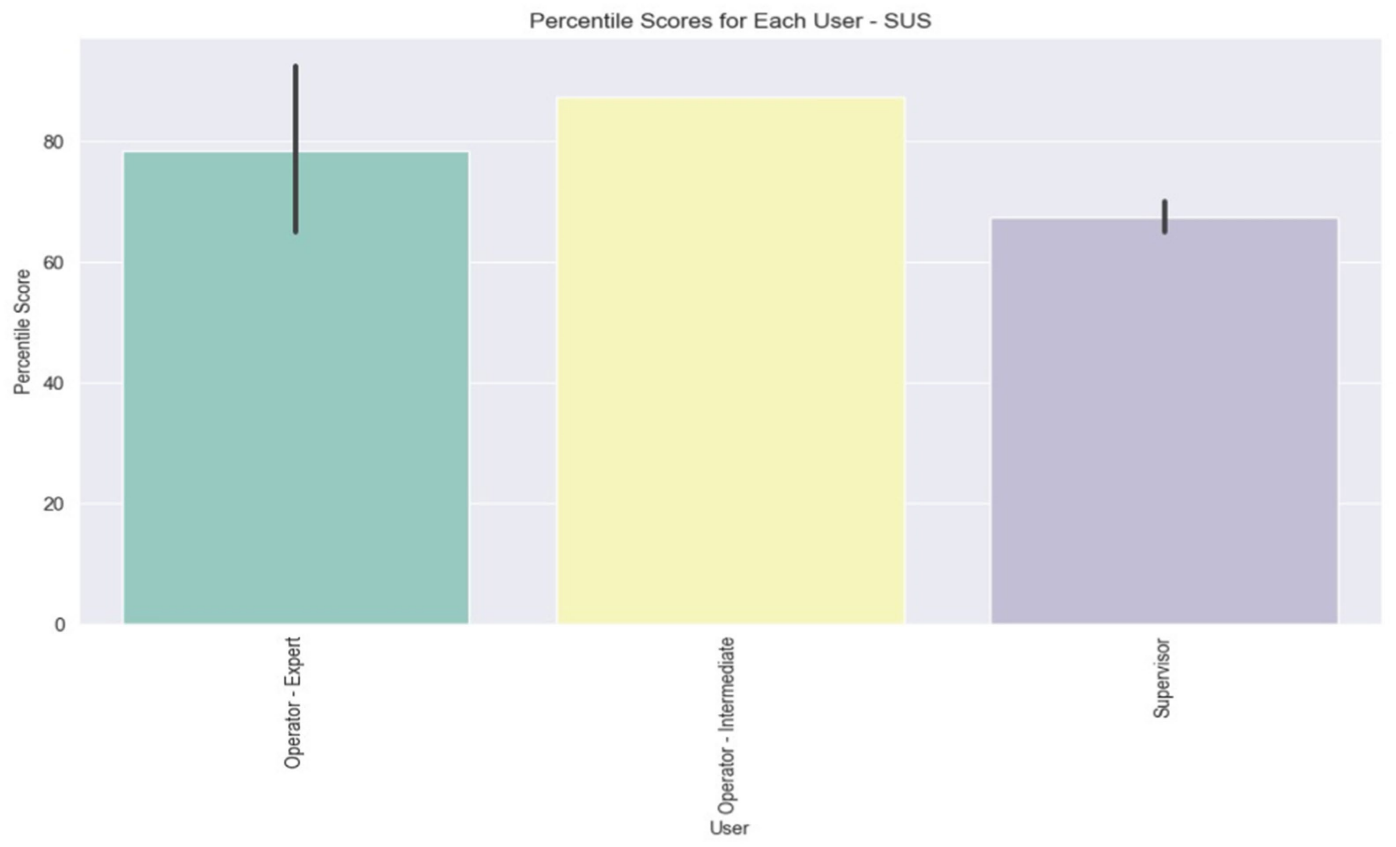
Average percentile SUS score for each user role.

In Figure 11, the average scores (ranging from 1 to 5) for each SUS question are illustrated. Notably, there is unanimity in the scores for questions 4, 7, and 10, which pertain, respectively, to the perceived need for technical support to use the system, the general consensus that most people would learn to use it quickly, and the anticipated necessity for users to learn a lot of things to operate the system. This uniformity in responses might stem from the users’ limited experience and interaction with the system. Moreover, it is evident that the lowest score was garnered by question 2, which probes into the perceived complexity of the system (Figure 12).

**Figure 13:**
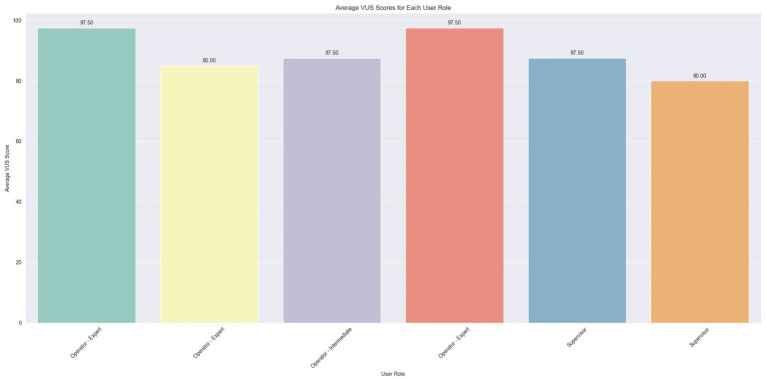
Average VUS score per user.

The same types of graphs are presented for the VUS scores. Table 5 cites the VUS questions. In Figure 13, the plot illustrates the average VUS score per user. Initially, it is observed that the VUS scores are generally higher compared to the SUS scores, with all users awarding an ‘Excellent’ rating (>80.3), except for one user whose score is marginally below, at 80. This suggests a generally favorable user response to voice usability as compared to overall system usability.

**Table 5:** VUS items.

Code	VUS Items
VUS_1	I thought the response from the voice assistant was easy to understand.
VUS_2	I thought the information provided by the voice assistant was not relevant to what I asked.
VUS_3	I felt the response from the voice assistant was sufficient.
VUS_4	I thought the voice assistant had difficulty in understanding what I asked it to do.
VUS_5	I felt the voice assistant enabled me to successfully complete my tasks when I required help.
VUS_6	I found it frustrating to use the voice assistant in a noisy and loud environment.
VUS_7	The voice assistant had all the functions and capabilities that I expected it to have.
VUS_8	I found it difficult to customize the voice assistant according to my needs and preferences.
VUS_9	Overall, I am satisfied with using the voice assistant.
VUS_10	I found the voice assistant difficult to use.

**Figure 14:**
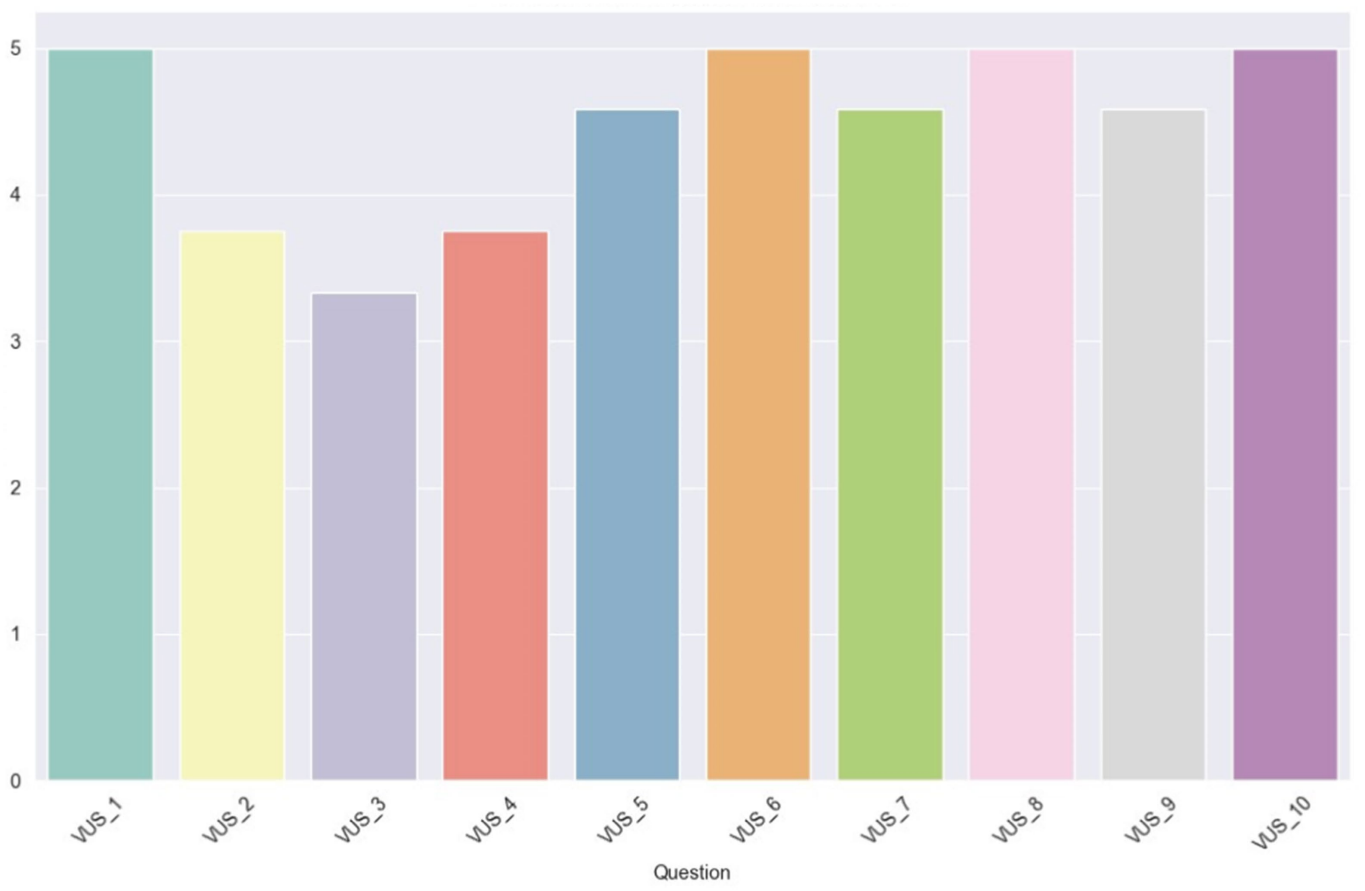
Average scores for VUS questions.

In Figure 14, the average scores (1–5) for each VUS question are depicted. Within this scale, four questions received unanimous ratings with the maximum score. These questions, numbered 1, 6, 8, and 10, address, respectively, the ease of understanding the voice, the level of frustration experienced using the assistant in a noisy environment, the difficulty in customizing the voice assistant, and the difficulty in using the voice assistant. It’s important to note that the questions with even numbering (6, 8, 10) are phrased with a negative sentiment, but the scores are inverted, meaning high values indicate a positive user experience. Additionally, all the scores are above the midpoint value of 3, suggesting a generally positive user response to these aspects of voice usability.

**Figure 15:**
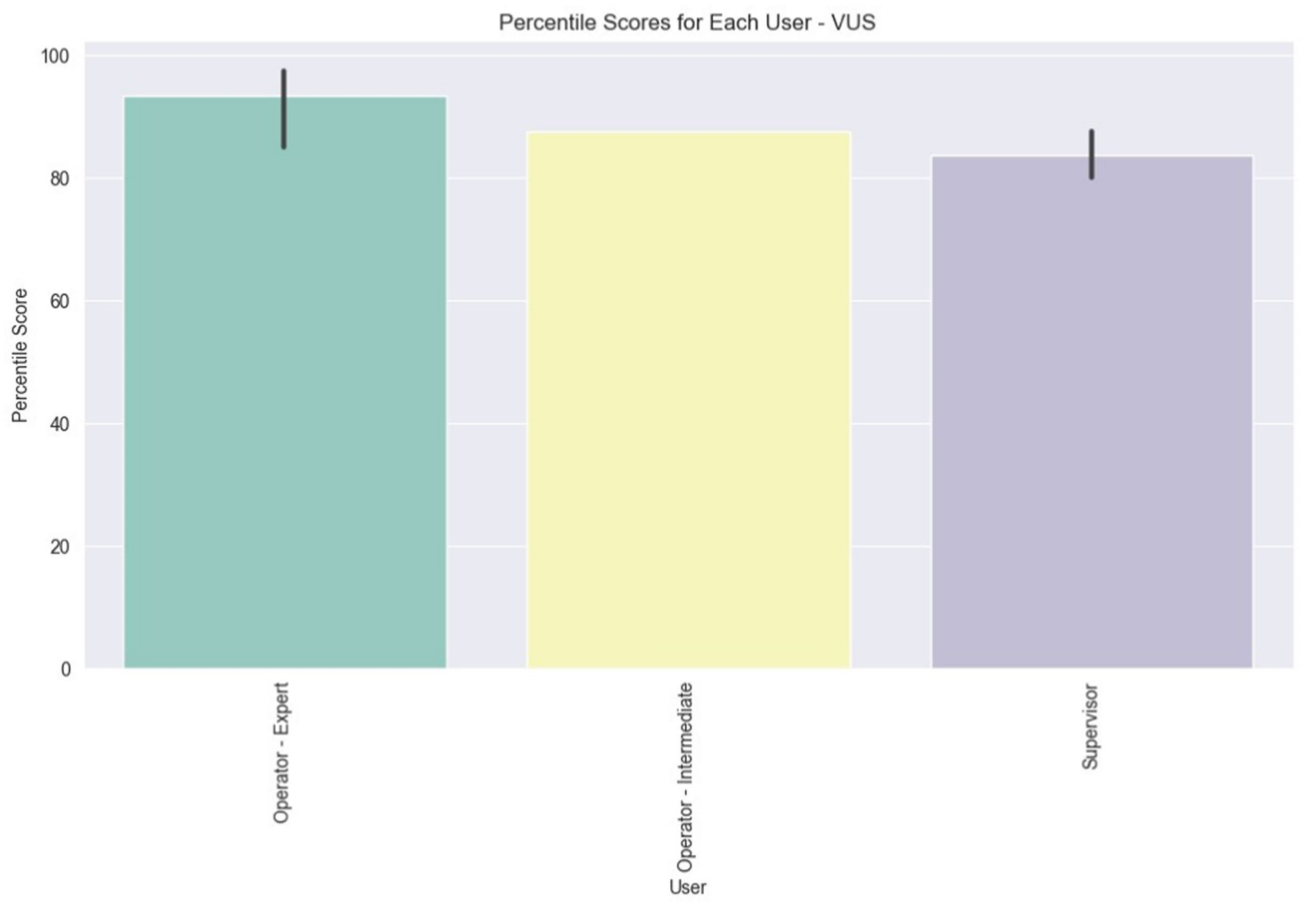
Average percentile VUS score for each user role.

In the final plot for VUS, depicted in Figure 15, the average scores per user role are illustrated. The initial observation drawn from the bar values is that all user roles have, on average, rated the VUS above 80. Additionally, there is a slight variation in the scores among the Expert users, and an even smaller variation among the Supervisors, as indicated by the vertical black lines atop the bar plots.

**Figure 16:**
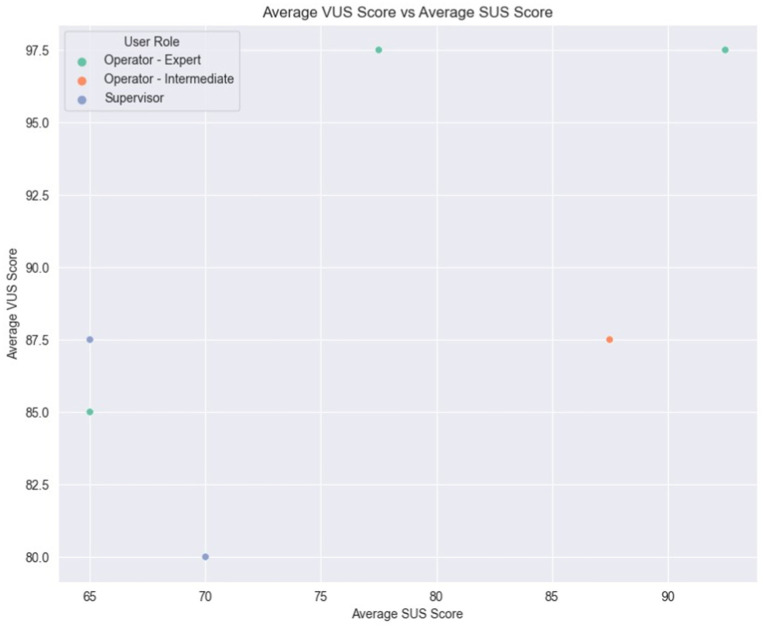
Average SUS vs. VUS scores.

Finally, Figure 16 illustrates a comparison of each user’s average scores between the SUS and VUS. As previously observed, variations in scores among the Expert Operators are evident, with two of them also recording the highest VUS scores. Moreover, the two Supervisors, along with one of the Expert Operators, appear to occupy the lower spectrum of scores for both scales, indicating a less favorable assessment of usability. Intriguingly, the Intermediate Operator showcased consistent ratings across the two scales, reflecting a uniform perception of both system and voice usability. This Figure also serves as a comparison between SUS and VUS, since the variation between them demonstrates the complexity while evaluating voice interface-based systems compared to GUI-based systems.

#### 5.3.3 Cognitive workload

**Figure 17:**
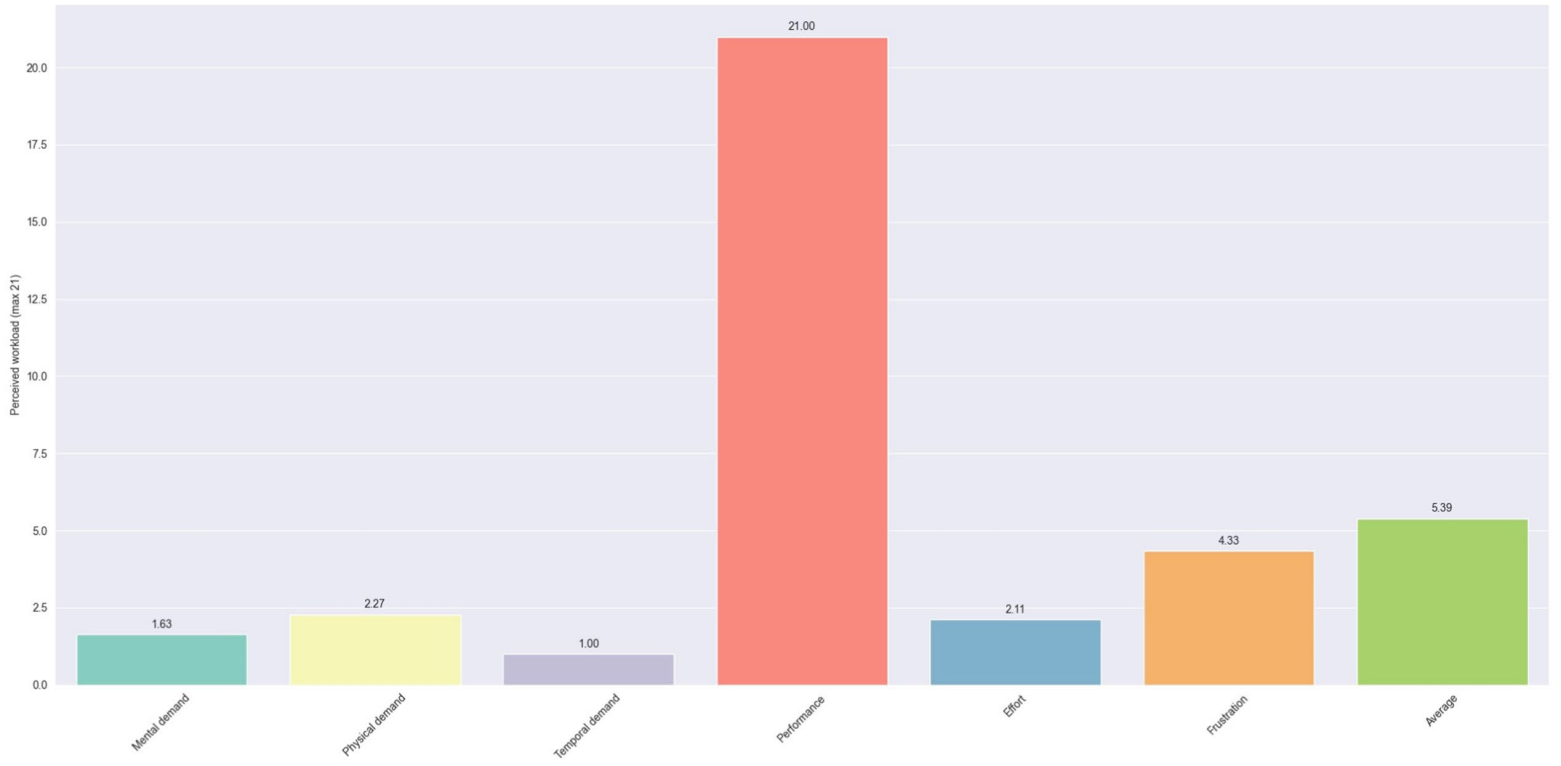
Perceived workload using NASA-TLX scores for the white goods production.

In Figure 17, the average scores for each question of the NASA-TLX questionnaire are presented. The most noticeable observation initially is the difference in score between the question regarding perceived performance and all other questions. The former has unanimously the highest score, reaching 21. In contrast, the questions about the perceived mental, physical, and temporal demands, along with the one regarding effort, all exhibit low scores under 2.5. This suggests that the system was not perceived as particularly demanding by the users. A slightly higher value is observed for perceived frustration, with a score of 4.33. This could potentially be derived from the users’ lack of familiarity with voice assistants.

#### 5.3.4 Technical robustness

In this sub-section, we present the results related to the technical validation of the solution’s main components. As far as the Analytics Service is concerned, we present metrics related to the accuracy of the AutoML models. The datasets used in these experiments were tabular containing datetime, numeric, categorical and text values. During preprocessing, text values were manually discarded due to inconsistencies and entries with missing values were removed. In this setting, we incorporated the Python libraries AutoKeras and FEDOT. The AutoKeras library generates Neural Networks (NN) and performs a Neural Architecture Search (NAS) guided by Bayesian Optimization and Gaussian processes. In our experiments, we employed the *StructuredDataClassifier* and the *StructuredDataRegressor* for the classification and regression tasks, respectively. They were also configured with the respective metrics and the maximum number of trails to perform was set to 20. For the FEDOT library, the *Fedot* pipeline was employed with task-specific parameters in each task. For the classification task, the task and problem types were set to *classification* while for the regression task, it was set to time series forecasting. In both tasks, the optimization was conducted simultaneously with the maximum number of tuning iterations set to 100. We implemented three models per algorithm: (i) an initial model trained with the 80% of the dataset; (ii) a retrained model, with the whole dataset; and (iii) a new model which executes the AutoML process on the whole dataset from the beginning. Moreover, it should be noted that the execution time depends on the pre-configured stopping conditions.

Table 6 presents the results of the Defect Group Prediction case. The data processing algorithms selected 6 features (i.e., Date Created, Product Type (SKU), Defect Source, Station ID, Part Family) and the performance of the AutoML models was evaluated by measuring F1-macro, F1-micro, Receiver Operating Characteristic Area Under Curve (ROC-AUC), and execution time. Table 7 presents the results of the Defective Orders Prediction case. The data processing algorithms summed the Defect Instances were on the attribute Date Created to produce the necessary timeseries, and the performance of the AutoML models was evaluated by measuring the Mean Square Error (MSE), the Mean Absolute Error (MAE), and the execution time. The FEDOT AutoML framework selected the Extreme Gradient Boosting (XGBoost) ML algorithm, while the AutoKeras AutoML framework selected the Neural Network ML algorithm.

**Table 6:** Results for the defect group prediction.

	AutoML models	Metrics			
		F1-macro	F1-micro	ROC-AUC	Execution time (s)
Initial models	FEDOT	0.5055	0.8363	0.9023	1212.50
	AutoKeras	0.4509	0.7813	0.7141	1019.81
Retrained models	FEDOT	0.4969	0.8368	0.9019	9.35
	AutoKeras	0.4510	0.7813	0.7141	0.58
New models	FEDOT	0.4909	0.8277	0.8722	91.96
	AutoKeras	0.4631	0.7681	0.7177	953.36

**Table 7:** Results for the defective orders prediction.

	AutoML models	Metrics		
		MSE	MAE	Execution time (s)
Initial models	FEDOT	0.2338	0.2017	101.88
	AutoKeras	0.0402	0.1624	187.39
Retrained models	FEDOT	0.1361	0.1002	0.32
	AutoKeras	0.0201	0.1082	2.57
New models	FEDOT	0.1391	0.0988	127.48
	AutoKeras	0.0191	0.1036	11.17

**Figure 18:**
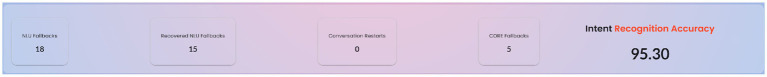
Intent recognition accuracy and fallback summary.

Throughout the evaluation period, as it was presented in Section 5.2, we were continuously improving the technical developments activities. In order to increase the intent recognition accuracy, we performed elimination of unusual words, streamline of sentences, usage of keywords, as well as usage of selection button on the tablet. These improvements drove the high accuracy in the user’s intention understanding. The intent recognition accuracy for the conversations in September 2023, with 402 conversation turns, was 95.30%, as depicted in Figure 18.

#### 5.3.5 Discussion on lessons learned and managerial implications

We performed workshops among the involved users and bilateral interviews in order to acquire qualitative feedback and to draw the main lessons learned. Below, we summarize the main conclusions derived from this procedure.

The solution provided the general validation of the cognitive worker assistance technology as one of the key enablers of a relevant change in the execution of industrial operations: the possibility of being assisted by an intelligent system, with a voice interface, which may drive the workers through the execution of complex operation, has been very appreciated by the users who confirmed the potential application not only in “off-line” working places, like laboratories or indirect activities (quality control, product repair, maintenance, logistics, material management) but also in “on-line” tasks execution like the quality control on the assembly where the pace has to be respected. A great advantage of the solution is the possibility to leave the hands free to execute tasks while the cognitive support understands the request, collects the right information, and provides it in a user-friendly format.

The possibility of having a unique interface to get access to all the information is very important as it boosts the efficiency in the navigation among the different systems of the IT landscape, ensuring the right information to the right person at the right time.

Mobile devices, such as tablets, are not the best equipment solution: tablets have to be managed by hand, interrupting task execution, and introducing inefficiency. The best solution has been identified in the usage of a headset and touch big screen, fixed in front of the operator to be easily seen and touched for the manual commands input, connected to a simple barcode scanner for SKU barcode reading. However, the adoption of wearable devices such as headsets introduces some constraints from a personnel management point of view: any wearable has to be strictly individual for safety and health reasons, and this may inflate the deployment cost of the solution in the factory.

The intensity of the information support and level of detail provided to the different people has been appreciated. However, the text referring to domain knowledge (e.g., quality checklists) includes long sentences, repetition, and wrong or incorrect words. This element highlighted the need to completely review the information content that has to be designed to be used in these types of digital systems and cannot replicate the structure that currently is used for the description on paper. The key reason behind this is that there was the need for a long knowledge acquisition period in order to simplify and create more robust relations for the syntax to be used.

The functionality to provide different services according to the user’s profile has been appreciated, as well as the deployment of the learning path through the different users’ skills profile (from novice to intermediate to expert). One remark has arisen by users related to this topic: the risk of having operators who can pass from novice to expert level faster than today could be penalized by the fact that these operators can be more “passive” towards the task execution as it is always suggested by the system. In this way, the risk of a passive approach will drive to the lack of “real expert” operators who are not able to execute without the system. The risk has to be mitigated by the real shift of the operator’s attention from the pure mechanical execution of tasks to the interpretation of the information provided. To achieve this objective, an intensive, focused training action has to be put in place, combined with the collection of ideas and rewarding management.

The quality risk assessment functionality has been much appreciated, and, for the first time in the factory, there is clear visibility of predictive quality analytics results. The good quality of the prediction has also been confirmed by users who got access to predictive results and confirmed them with real experience on the various defective parts and products in the actual production process.

The huge amount of data with poor quality is one of the most common cases in the IT landscape of manufacturing companies, which, for years, have been collecting data from the shop floor without real and effective usage of them. This element forces the IT organization to create a stronger link with the shop floor to deploy a more effective solution: for Whirlpool this will pass through a redesign of the data architecture in the cloud in order to more effectively and efficiently support the analytics functionalities deployment. Therefore, the need for a deep review of the actual data management strategy has arisen: the need to count on a high-quality database, real-time updated, and designed to be efficiently integrated with digital functionalities proved to be one of the higher priorities in digital transformation. Whirlpool identified that they need to be based upon a completely different quality control data model.

A focused change management strategy has been defined, and it has been used as the backbone for the creation and deployment of communication and training actions not only toward the involved users but also engaging the overall factory organization at different levels. These activities put evidence on the need for great attention on how these types of technology are presented and deployed to the impacted population in order to create the right level of awareness on potential and risks and enable the real adoption of the solution.

The adoption of the solution seriously and structurally faces privacy and GDPR compliance management in the deployment of voice assistance applications. The execution of the change management actions and the “privacy by design” effort spent for system development and consensus form finalization, put evidence on the real poor level of awareness of people on the potential impact of this technology in the working environment, and, in parallel, also at home and in their personal life.

### 5.4 Discussion on generalizability criteria

The evaluation results showed that the integration of voice assistance technology with AutoML has the potential to significantly contribute to the increase of operational efficiency. In order to apply the proposed approach to different manufacturing use cases, the challenges of AutoML when it uses a voice interface for user interaction should be taken into account. Therefore, the following criteria need to be considered by manufacturers, software developers, data scientists, and practitioners:

**Accuracy:** It should achieve high prediction accuracy. It should be taken into account that higher accuracy may need more computational resources.

**Efficiency:** It should be efficient in terms of time and computational resources. The automated processes should be designed to optimize the use of resources and reduce the overall time required to train models and make predictions. This particularly applies to applications that are critical in terms of time (high sampling time). In these cases, prediction accuracy may negatively be affected.

**Scalability:** It should be capable of scaling up to handle large datasets and complex ML tasks and pipelines. They should be able to handle increasing amounts of data and computational demands without compromising performance or efficiency.

**Flexibility:** It should provide flexibility by supporting several ML algorithms. It should allow users to experiment with different approaches and customize the automated processes according to their specific requirements and preferences.

**Explainability:** It should be accompanied with explainability mechanisms, suitable for voice interfaces, in order to enable the human to understand the inner working and the outcomes of the “black-box” AutoML pipelines.

**Transparency:** It should be able to provide evidence about the ML models and pipelines that were used for generating some specific insights in order to contribute to the increase of user trust.

**Robustness:** It should be robust against unexpected inputs and real-world industrial data challenges, such as noise, missing values, and imbalances. This is particularly important in safety-critical tasks.

**Adaptability:** It should be capable of adapting across various domains and use cases covering diverse business requirements and manufacturing operations.

**Integration with existing IT systems:** It should incorporate interfaces, based on related standards, for facilitating the integration with existing IT infrastructures and production systems, i.e., legacy systems, Enterprise Resource Planning (ERP), Manufacturing Execution Systems (MES), quality management systems, etc., according to the application domain and the manufacturing operation at hand. It should also support both a cloud-based integration and an on-premise integration, providing sufficient documentation.

**Cost-effectiveness:** It should minimize the additional effort required for installing and configuring the solution.

**Compliance with ethical and regulatory standards:** It should be compliant with trustworthy and ethical Artificial Intelligence (AI) principles, taking into account related regulations, such as ALTAI and EU AI Act.

## 6 Conclusion and future work

Augmented intelligence puts together human and artificial agents to create a socio-technological system so that they co-evolve by learning and optimizing decisions through intuitive interfaces, such as conversational, voice-enabled interfaces. However, existing research works on voice assistants rely on knowledge management and simulation methods instead of data-driven algorithms in order to take advantage of the large amounts of data existing in modern manufacturing environments. In addition, practical application and evaluation in real-life scenarios are scarce and limited in scope due to the aforementioned challenges.

In this paper, we proposed the integration of voice assistance technology with AutoML in order to enable the realization of the augmented intelligence paradigm in the context of Industry 5.0. AutoML automates the building and deployment of ML pipelines without requiring ML knowledge, while the voice interface exposes the data analytics outcomes to the user in an intuitive way. On the other hand, the user is able to interact with the assistant, and consequently with the ML models, through voice in order to receive immediate insights while performing their task. The proposed approach was evaluated in a real manufacturing environment. We followed a structured evaluation methodology and analyzed the results, which demonstrate the effectiveness of our proposed approach.

Our future work will move towards the following directions: (i) We will extend the proposed solution by utilizing LLM for user interaction in order to enhance the scalability and adaptability of the DIA to various industrial settings with less human effort at the design time of the solution; (ii) We will evaluate the proposed solution in additional real-life manufacturing scenarios from various sectors taking into account the generalizability criteria that were derived from the current research work.; and, (iii) We will focus on how transparency and explainability approaches for AutoML can be incorporated when there is a voice user interface instead of a GUI.

## Data Availability

The datasets for this article are not publicly available due to legal and privacy-related restrictions in relation to confidential human data. Requests to access the datasets should be directed to the corresponding author.

## Footnotes

^1^ https://altai.insight-centre.org/

## Supplementary material

SUPPLEMENTARY TABLE S1 Resulting ALTAI recommendations for each out of the 7 requirements.
