# Supplementary material for: Augmented intelligence with voice assistance and automated machine learning in Industry 5.0
Source: Front Artif Intell. 2025 Mar 4;8:1538840. doi: 10.3389/frai.2025.1538840 (PMC11913813; doi:10.3389/frai.2025.1538840)
Supplement: SUPPLEMENTARY TABLE S1 — Resulting ALTAI recommendations for each out of the 7 requirements. [file Table_1.docx]

Supplementary Table 1

| **1** | **Human agency and oversight** |
| --- | --- |
| *No recommendation for this requirement.* | |
| **2** | **Technical robustness and safety** |
| *No recommendation for this requirement.* | |
| **3** | **Privacy and Data Governance** |
| 1. Whenever possible and relevant, align the AI-system with relevant standards (e.g. ISO, IEEE) or widely adopted protocols for data management and governance. | |
| **4** | **Transparency** |
| *No recommendation for this requirement.* | |
| **5** | **Diversity, non-discrimination and fairness** |
| 1. Your definition of fairness should be commonly used and should be implemented in any phase of the process of setting up the AI system. 2. Consider other definitions of fairness before choosing one. 3. Consult with the impacted communities about the correct definition of fairness. 4. Ensure a quantitative analysis to measure and test the applied definition of fairness. 5. Establish mechanisms to ensure fairness in your AI system. 6. You should assess whether the AI system's user interface is usable by those with special needs or disabilities or those at risk of exclusion. 7. You should assess the risk of the possible unfairness onto the end-user's communities. | |
| **6** | **Societal and environmental well-being** |
| *No recommendation for this requirement.* | |
| **7** | **Accountability** |
| 1. 3rd party auditing can contribute to generate trust in the technology and the product itself. Additionally, it is a strong indication of adhering to industrial standards. 2. If AI systems are increasingly used for decision support or for taking decisions them-selves, it has to be made sure these systems are fair in their impact on people’s lives, that they are in line with values that should not be compromised and able to act accordingly, and that suitable accountability processes can ensure this. 3. A risk management process should include new findings since initial assumptions about the likelihood of occurrence for a specific risk might be faulty. 4. Acknowledging that redress is needed when incorrect predictions can cause adverse impacts to individuals is key to ensure trust. | |
